# *N*-(2,6-Dichloro­phen­yl)benzene­sulfonamide

**DOI:** 10.1107/S160053681004420X

**Published:** 2010-11-06

**Authors:** P. G. Nirmala, Sabine Foro, B. Thimme Gowda, Hartmut Fuess

**Affiliations:** a Department of Chemistry, Mangalore University, Mangalagangotri 574 199, Mangalore, India; b Institute of Materials Science, Darmstadt University of Technology, Petersenstrasse 23, D-64287 Darmstadt, Germany

## Abstract

In the title compound, C_12_H_9_Cl_2_NO_2_S, the mol­ecule is bent at the S atom with a C—SO_2_—NH—C torsion angle of 82.5 (2)°. The benzene rings are tilted relative to each other by 43.5 (1)°. The crystal structure features chains linked by N—H⋯O hydrogen bonds.

## Related literature

For our study of the effect of substituents on the structures of *N*-(ar­yl)aryl­sulfonamides, see: Gowda *et al.* (2008*a* ▶,*b* ▶, 2010 ▶). For related structures, see: Gelbrich *et al.* (2007 ▶); Perlovich *et al.* (2006 ▶).
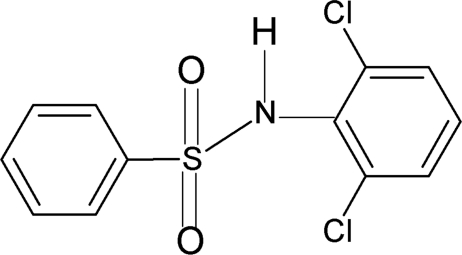


## Experimental

### 

#### Crystal data

C_12_H_9_Cl_2_NO_2_S
*M* *_r_* = 302.16
Monoclinic, 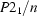

*a* = 5.059 (1) Å
*b* = 17.143 (4) Å
*c* = 15.351 (3) Å
β = 90.43 (2)°
*V* = 1331.3 (5) Å^3^
*Z* = 4
Mo *K*α radiation
μ = 0.64 mm^−1^
*T* = 293 K
0.20 × 0.10 × 0.10 mm

#### Data collection

Oxford Diffraction Xcalibur diffractometer with a Sapphire CCD detector
Absorption correction: multi-scan (*CrysAlis RED*; Oxford Diffraction, 2009 ▶) *T*_min_ = 0.883, *T*_max_ = 0.939
4603 measured reflections
2423 independent reflections
1389 reflections with *I* > 2σ(*I*)
*R*_int_ = 0.031

#### Refinement

*R*[*F* ^2^ > 2σ(*F* ^2^)] = 0.040
*wR*(*F* ^2^) = 0.088
*S* = 0.85
2423 reflections
166 parameters
1 restraint
H atoms treated by a mixture of independent and constrained refinement
Δρ_max_ = 0.26 e Å^−3^
Δρ_min_ = −0.29 e Å^−3^

### 

Data collection: *CrysAlis CCD* (Oxford Diffraction, 2009 ▶); cell refinement: *CrysAlis RED* (Oxford Diffraction, 2009 ▶); data reduction: *CrysAlis RED*; program(s) used to solve structure: *SHELXS97* (Sheldrick, 2008 ▶); program(s) used to refine structure: *SHELXL97* (Sheldrick, 2008 ▶); molecular graphics: *PLATON* (Spek, 2009 ▶); software used to prepare material for publication: *SHELXL97*.

## Supplementary Material

Crystal structure: contains datablocks I, global. DOI: 10.1107/S160053681004420X/bq2247sup1.cif

Structure factors: contains datablocks I. DOI: 10.1107/S160053681004420X/bq2247Isup2.hkl

Additional supplementary materials:  crystallographic information; 3D view; checkCIF report

## Figures and Tables

**Table 1:** Hydrogen-bond geometry (Å, °)

*D*—H⋯*A*	*D*—H	H⋯*A*	*D*⋯*A*	*D*—H⋯*A*
N1—H1N⋯O1^i^	0.83 (2)	2.21 (2)	3.027 (3)	166 (3)

Symmetry code: (i) 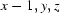.

**Fig. 1.**
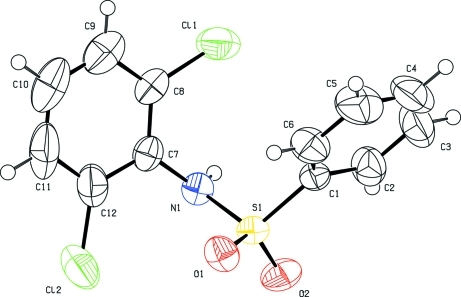
Molecular structure of (I), showing the atom labeling scheme and displacement ellipsoids are drawn at the 50% probability level.

**Fig. 2.**
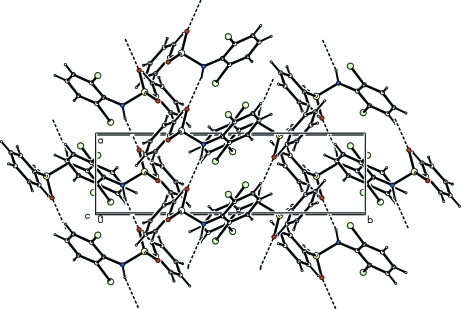
Molecular packing of (I) with hydrogen bonding shown as dashed lines.

## supplementary crystallographic information

### Comment

As part of a study of substituent effects on the structures of *N*-(aryl)arylsulfonamides (Gowda *et al.* , 2008*a*, *b*, 2010), the structure of *N*-(2,6-dichlorophenyl)- benzenesulfonamide (I) has been determined (Fig. 1). The molecule is bent at the *S* atom with the C—SO_2_—NH—C torsion angle of 82.5 (2)°, compared to the values of -62.1 (3)° and 60.7 (3)°, in the two molecules of *N*-(2,4-dichlorophenyl)benzenesulfonamide (II) (Gowda *et al.*, 2010), -68.1 (3)° in *N*-(3,5-dichlorophenyl)- benzenesulfonamide (III) (Gowda *et al.*, 2008*b*) and -78.7 (2)° in *N*-(2,6-dimethylphenyl)benzenesulfonamide (IV) (Gowda *et al.*, 2008*a*).

The sulfonyl benzene and the aniline benzene rings in (I) are tilted relative to each other by 43.5 (1)°, compared to the values of 70.8 (1)° (molecule 1) and 74.8 (1)° (molecule 2) in (II), 57.0 (1)° in (III) and 44.9 (1)° in (IV). The other bond parameters in (I) are similar to those observed in (II)-(IV) and other aryl sulfonamides (Perlovich *et al.*, 2006; Gelbrich *et al.*, 2007).

The crystal packing of molecules in (I) *via* N—H···O(S) hydrogen bonds (Table 1) is shown in Fig. 2.

### Experimental

The solution of benzene (10 ml) in chloroform (40 ml) was treated dropwise with chlorosulfonic acid (25 ml) at 0 ° C. After the initial evolution of hydrogen chloride subsided, the reaction mixture was brought to room temperature and poured into crushed ice in a beaker. The chloroform layer was separated, washed with cold water and allowed to evaporate slowly. The residual benzenesulfonylchloride was treated with 2,6-dichloroaniline in the stoichiometric amounts and boiled for ten minutes. The reaction mixture was then cooled to room temperature and added to ice cold water (100 ml). The resultant solid *N*-(2,6-dichlorophenyl)benzenesulfonamide was filtered under suction and washed thoroughly with cold water. It was then recrystallized to constant melting point from dilute ethanol. The purity of the compound was checked and characterized by recording its infrared and NMR spectra. The prism like colorless single crystals used in X-ray diffraction studies were grown in ethanolic solution by evaporating it at room temperature.

### Refinement

The H atom of the NH group was located in a difference map and later restrained to the distance N—H = 0.86 (2) Å. The other H atoms were positioned with idealized geometry using a riding model with C—H = 0.93 Å. All H atoms were refined with isotropic displacement parameters (set to 1.2 times of the *U*_eq_ of the parent atom).

### Crystal data

**Table:**

C_12_H_9_Cl_2_NO_2_S	*F*(000) = 616
*M**_r_* = 302.16	*D*_x_ = 1.508 Mg m^−^^3^
Monoclinic, *P*2_1_/*n*	Mo *K*α radiation, λ = 0.71073 Å
Hall symbol: -P 2yn	Cell parameters from 1612 reflections
*a* = 5.059 (1) Å	θ = 2.9–27.9°
*b* = 17.143 (4) Å	µ = 0.64 mm^−^^1^
*c* = 15.351 (3) Å	*T* = 293 K
β = 90.43 (2)°	Prism, colourless
*V* = 1331.3 (5) Å^3^	0.20 × 0.10 × 0.10 mm
*Z* = 4	

### Data collection

**Table:**

Oxford Diffraction Xcalibur diffractometer with a Sapphire CCD detector	2423 independent reflections
Radiation source: fine-focus sealed tube	1389 reflections with *I* > 2σ(*I*)
graphite	*R*_int_ = 0.031
Rotation method data acquisition using ω and phi scans	θ_max_ = 25.4°, θ_min_ = 2.9°
Absorption correction: multi-scan (*CrysAlis RED*; Oxford Diffraction, 2009)	*h* = −5→6
*T*_min_ = 0.883, *T*_max_ = 0.939	*k* = −20→20
4603 measured reflections	*l* = −18→17

### Refinement

**Table:**

Refinement on *F*^2^	Primary atom site location: structure-invariant direct methods
Least-squares matrix: full	Secondary atom site location: difference Fourier map
*R*[*F*^2^ > 2σ(*F*^2^)] = 0.040	Hydrogen site location: inferred from neighbouring sites
*wR*(*F*^2^) = 0.088	H atoms treated by a mixture of independent and constrained refinement
*S* = 0.85	*w* = 1/[σ^2^(*F*_o_^2^) + (0.0454*P*)^2^] where *P* = (*F*_o_^2^ + 2*F*_c_^2^)/3
2423 reflections	(Δ/σ)_max_ = 0.001
166 parameters	Δρ_max_ = 0.26 e Å^−^^3^
1 restraint	Δρ_min_ = −0.28 e Å^−^^3^

### Special details

**Table:**

Experimental. Absorption correction: CrysAlis RED (Oxford Diffraction, 2009) Empirical absorption correction using spherical harmonics, implemented in SCALE3 ABSPACK scaling algorithm.
Geometry. All e.s.d.'s (except the e.s.d. in the dihedral angle between two l.s. planes) are estimated using the full covariance matrix. The cell e.s.d.'s are taken into account individually in the estimation of e.s.d.'s in distances, angles and torsion angles; correlations between e.s.d.'s in cell parameters are only used when they are defined by crystal symmetry. An approximate (isotropic) treatment of cell e.s.d.'s is used for estimating e.s.d.'s involving l.s. planes.
Refinement. Refinement of *F*^2^ against ALL reflections. The weighted *R*-factor *wR* and goodness of fit *S* are based on *F*^2^, conventional *R*-factors *R* are based on *F*, with *F* set to zero for negative *F*^2^. The threshold expression of *F*^2^ > σ(*F*^2^) is used only for calculating *R*-factors(gt) *etc*. and is not relevant to the choice of reflections for refinement. *R*-factors based on *F*^2^ are statistically about twice as large as those based on *F*, and *R*- factors based on ALL data will be even larger.

### Fractional atomic coordinates and isotropic or equivalent isotropic displacement parameters (Å^2^)

**Table:**

	*x*	*y*	*z*	*U*_iso_*/*U*_eq_	
C1	−0.0324 (5)	0.27365 (16)	0.83013 (17)	0.0403 (7)	
C2	−0.2352 (5)	0.22113 (18)	0.8401 (2)	0.0619 (9)	
H2	−0.3479	0.2095	0.7937	0.074*	
C3	−0.2686 (8)	0.1860 (2)	0.9200 (3)	0.0893 (13)	
H3	−0.4066	0.1509	0.9277	0.107*	
C4	−0.1019 (9)	0.2020 (3)	0.9883 (3)	0.0926 (14)	
H4	−0.1254	0.1775	1.0417	0.111*	
C5	0.0986 (8)	0.2541 (3)	0.9776 (2)	0.0850 (12)	
H5	0.2113	0.2654	1.0241	0.102*	
C6	0.1354 (6)	0.2900 (2)	0.89885 (19)	0.0610 (9)	
H6	0.2732	0.3253	0.8918	0.073*	
C7	−0.0445 (5)	0.46992 (17)	0.76239 (19)	0.0444 (7)	
C8	−0.1209 (5)	0.49608 (17)	0.8443 (2)	0.0507 (8)	
C9	−0.0221 (7)	0.5630 (2)	0.8804 (3)	0.0782 (11)	
H9	−0.0799	0.5794	0.9348	0.094*	
C10	0.1620 (9)	0.6057 (2)	0.8364 (4)	0.0954 (14)	
H10	0.2337	0.6503	0.8616	0.115*	
C11	0.2409 (8)	0.5827 (2)	0.7553 (4)	0.0965 (14)	
H11	0.3652	0.6121	0.7254	0.116*	
C12	0.1356 (6)	0.5151 (2)	0.7169 (2)	0.0657 (9)	
N1	−0.1455 (4)	0.40045 (13)	0.72490 (14)	0.0410 (6)	
H1N	−0.306 (3)	0.3913 (15)	0.7287 (17)	0.049*	
O1	0.2938 (3)	0.33879 (11)	0.72465 (12)	0.0504 (5)	
O2	−0.0928 (4)	0.26891 (12)	0.66336 (12)	0.0588 (6)	
Cl1	−0.35415 (17)	0.44425 (5)	0.90206 (6)	0.0749 (3)	
Cl2	0.2244 (2)	0.49191 (6)	0.61194 (7)	0.1076 (4)	
S1	0.02094 (12)	0.31832 (4)	0.72850 (4)	0.0389 (2)	

### Atomic displacement parameters (Å^2^)

**Table:**

	*U*^11^	*U*^22^	*U*^33^	*U*^12^	*U*^13^	*U*^23^
C1	0.0385 (14)	0.0401 (17)	0.0423 (16)	0.0057 (13)	0.0055 (12)	−0.0008 (14)
C2	0.0525 (18)	0.060 (2)	0.073 (2)	−0.0069 (17)	0.0083 (16)	0.0062 (19)
C3	0.087 (3)	0.076 (3)	0.106 (3)	0.002 (2)	0.040 (3)	0.032 (3)
C4	0.116 (4)	0.096 (4)	0.066 (3)	0.038 (3)	0.040 (3)	0.033 (3)
C5	0.102 (3)	0.104 (3)	0.049 (2)	0.025 (3)	−0.009 (2)	0.009 (2)
C6	0.067 (2)	0.073 (2)	0.0428 (18)	−0.0008 (18)	−0.0073 (15)	0.0026 (18)
C7	0.0396 (15)	0.0379 (17)	0.056 (2)	0.0038 (14)	−0.0061 (14)	0.0021 (16)
C8	0.0505 (17)	0.0408 (19)	0.061 (2)	0.0048 (15)	−0.0099 (15)	−0.0073 (16)
C9	0.089 (3)	0.059 (3)	0.086 (3)	0.006 (2)	−0.016 (2)	−0.016 (2)
C10	0.097 (3)	0.049 (3)	0.140 (4)	−0.012 (2)	−0.024 (3)	−0.013 (3)
C11	0.084 (3)	0.050 (3)	0.156 (4)	−0.016 (2)	0.010 (3)	0.028 (3)
C12	0.064 (2)	0.049 (2)	0.084 (3)	0.0000 (18)	0.0077 (18)	0.018 (2)
N1	0.0307 (12)	0.0445 (15)	0.0478 (14)	0.0024 (11)	−0.0032 (11)	0.0009 (12)
O1	0.0306 (10)	0.0601 (14)	0.0606 (13)	0.0023 (9)	0.0049 (8)	0.0080 (11)
O2	0.0684 (12)	0.0622 (14)	0.0456 (12)	0.0019 (11)	−0.0103 (10)	−0.0189 (11)
Cl1	0.0761 (6)	0.0825 (7)	0.0664 (6)	0.0001 (5)	0.0221 (4)	−0.0150 (5)
Cl2	0.1395 (9)	0.0857 (8)	0.0984 (8)	0.0114 (7)	0.0556 (7)	0.0354 (7)
S1	0.0372 (4)	0.0437 (4)	0.0358 (4)	0.0029 (3)	−0.0003 (3)	−0.0040 (4)

### Geometric parameters (Å, °)

**Table:**

C1—C2	1.374 (4)	C7—N1	1.417 (3)
C1—C6	1.378 (4)	C8—C9	1.368 (4)
C1—S1	1.761 (3)	C8—Cl1	1.728 (3)
C2—C3	1.378 (4)	C9—C10	1.367 (5)
C2—H2	0.9300	C9—H9	0.9300
C3—C4	1.367 (5)	C10—C11	1.368 (5)
C3—H3	0.9300	C10—H10	0.9300
C4—C5	1.363 (5)	C11—C12	1.402 (5)
C4—H4	0.9300	C11—H11	0.9300
C5—C6	1.370 (4)	C12—Cl2	1.723 (4)
C5—H5	0.9300	N1—S1	1.641 (2)
C6—H6	0.9300	N1—H1N	0.832 (16)
C7—C12	1.388 (4)	O1—S1	1.4259 (17)
C7—C8	1.392 (4)	O2—S1	1.4284 (19)
			
C2—C1—C6	120.3 (3)	C7—C8—Cl1	119.6 (2)
C2—C1—S1	120.2 (2)	C10—C9—C8	119.8 (4)
C6—C1—S1	119.5 (2)	C10—C9—H9	120.1
C1—C2—C3	118.8 (3)	C8—C9—H9	120.1
C1—C2—H2	120.6	C9—C10—C11	120.0 (4)
C3—C2—H2	120.6	C9—C10—H10	120.0
C4—C3—C2	121.0 (4)	C11—C10—H10	120.0
C4—C3—H3	119.5	C10—C11—C12	120.5 (4)
C2—C3—H3	119.5	C10—C11—H11	119.8
C5—C4—C3	119.7 (4)	C12—C11—H11	119.8
C5—C4—H4	120.2	C7—C12—C11	119.9 (3)
C3—C4—H4	120.2	C7—C12—Cl2	121.2 (3)
C4—C5—C6	120.4 (4)	C11—C12—Cl2	118.8 (3)
C4—C5—H5	119.8	C7—N1—S1	121.60 (17)
C6—C5—H5	119.8	C7—N1—H1N	118.7 (19)
C5—C6—C1	119.8 (3)	S1—N1—H1N	109.7 (19)
C5—C6—H6	120.1	O1—S1—O2	120.14 (11)
C1—C6—H6	120.1	O1—S1—N1	106.50 (12)
C12—C7—C8	117.5 (3)	O2—S1—N1	106.33 (12)
C12—C7—N1	120.1 (3)	O1—S1—C1	107.38 (12)
C8—C7—N1	122.4 (3)	O2—S1—C1	107.38 (13)
C9—C8—C7	122.2 (3)	N1—S1—C1	108.71 (12)
C9—C8—Cl1	118.2 (3)		
			
C6—C1—C2—C3	0.7 (4)	N1—C7—C12—C11	−178.2 (3)
S1—C1—C2—C3	178.8 (3)	C8—C7—C12—Cl2	−175.1 (2)
C1—C2—C3—C4	−0.8 (5)	N1—C7—C12—Cl2	4.2 (4)
C2—C3—C4—C5	0.8 (6)	C10—C11—C12—C7	−1.8 (5)
C3—C4—C5—C6	−0.6 (6)	C10—C11—C12—Cl2	175.8 (3)
C4—C5—C6—C1	0.4 (6)	C12—C7—N1—S1	81.8 (3)
C2—C1—C6—C5	−0.5 (5)	C8—C7—N1—S1	−98.9 (3)
S1—C1—C6—C5	−178.5 (3)	C7—N1—S1—O1	−32.9 (2)
C12—C7—C8—C9	−0.9 (4)	C7—N1—S1—O2	−162.2 (2)
N1—C7—C8—C9	179.7 (3)	C7—N1—S1—C1	82.5 (2)
C12—C7—C8—Cl1	177.5 (2)	C2—C1—S1—O1	−153.7 (2)
N1—C7—C8—Cl1	−1.9 (4)	C6—C1—S1—O1	24.4 (3)
C7—C8—C9—C10	−1.3 (5)	C2—C1—S1—O2	−23.2 (3)
Cl1—C8—C9—C10	−179.8 (3)	C6—C1—S1—O2	154.9 (2)
C8—C9—C10—C11	2.0 (6)	C2—C1—S1—N1	91.5 (2)
C9—C10—C11—C12	−0.5 (6)	C6—C1—S1—N1	−90.4 (2)
C8—C7—C12—C11	2.5 (4)		

### Hydrogen-bond geometry (Å, °)

**Table:**

*D*—H···*A*	*D*—H	H···*A*	*D*···*A*	*D*—H···*A*
N1—H1N···O1^i^	0.83 (2)	2.21 (2)	3.027 (3)	166 (3)

Symmetry codes: (i) *x*−1, *y*, *z*.
